# Characteristics, capability, and origin of shale gas desorption of the Longmaxi Formation in the southeastern Sichuan Basin, China

**DOI:** 10.1038/s41598-018-37782-2

**Published:** 2019-01-31

**Authors:** Xianglu Tang, Zhenxue Jiang, Shu Jiang, Lijun Cheng, Ningning Zhong, Ling Tang, Jiaqi Chang, Wen Zhou

**Affiliations:** 1 State Key Laboratory of Petroleum Resources and Prospecting, China University of Petroleum, Beijing, 102249 China; 2 Unconventional Petroleum Research Institute, China University of Petroleum, Beijing, 102249 China; 3 Energy and Geoscience Institute, University of Utah, Salt Lake City, Utah 84108 USA; 4 Chongqing Institute of Geology and Mineral Resources, Chongqing, 400042 China

## Abstract

Shale gas desorption and loss is a serious and common phenomenon in the Sichuan Basin. The characteristics, capability, and origin of shale gas desorption are significant for understanding the shale gas reservoir accumulation mechanism and guiding shale gas exploration. The shale gas of the Longmaxi Formation in the southeastern Sichuan Basin was studied based on a shale gas desorption simulation experiment, combined with mineral composition, total organic carbon, specific surface area, isothermal adsorption, and scanning electron microscope (SEM) data. Here, the shale gas desorption capability was quantitatively evaluated, and its controlling factors are discussed. The results show that the shale gas desorption process within the Longmaxi Formation varies significantly. The total time of the desorption process varies from 600 min to 4400 min, and it mainly occurs by the 98 °C desorption stage. The desorption capability of the lower Formation is markedly weaker than that of the upper Formation, and it is mainly determined by the shale properties. Organic matter (OM) is the most important controlling factor. As the OM content increases, the specific surface area, methane adsorption capacity, and OM pores increase, leading to a rapid decrease in shale gas desorption capability. In addition, feldspar exhibits a positive correlation with shale gas desorption capability due to its large pores but low specific surface area.

## Introduction

**Figure 1:**
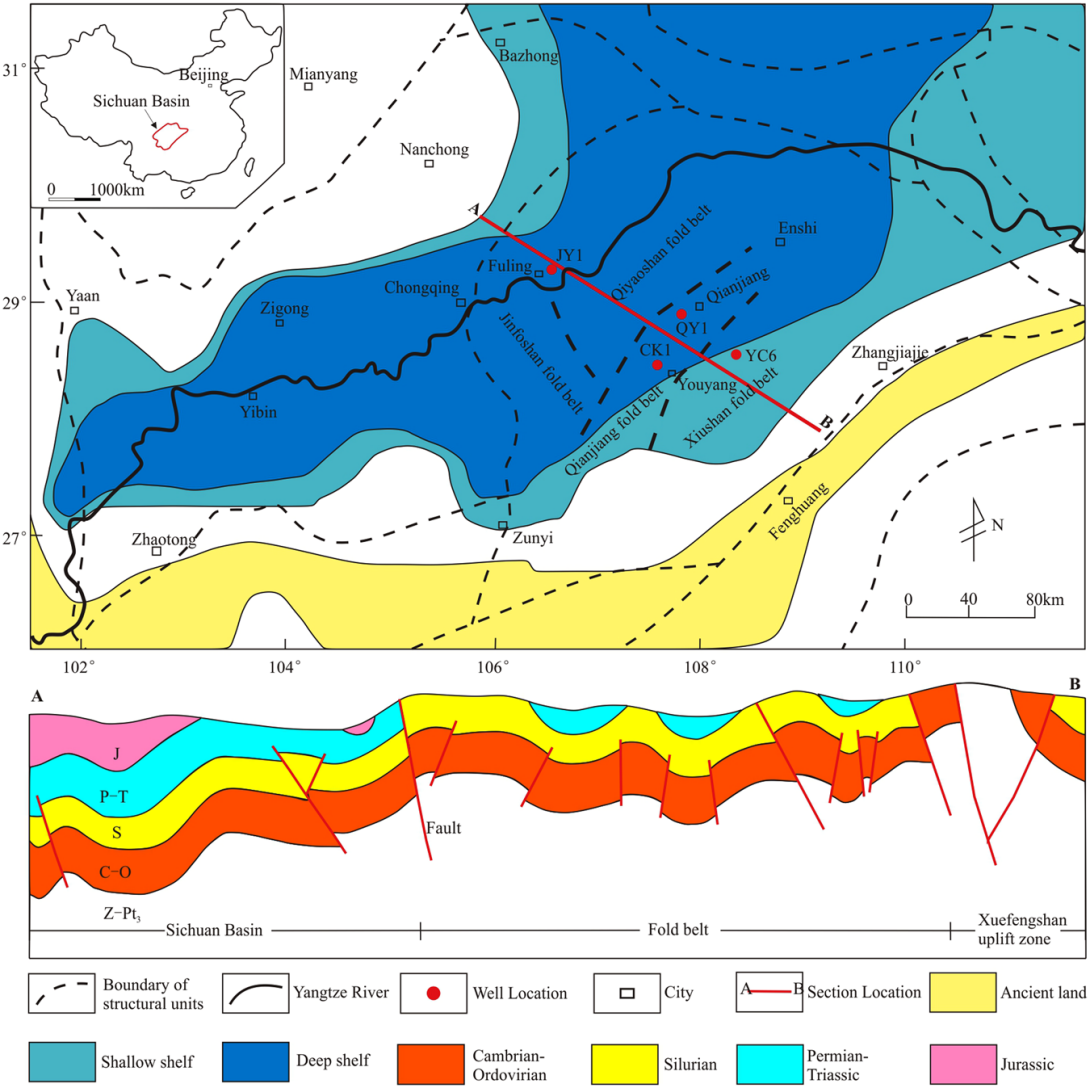
Location and tectonic setting of the southeastern Sichuan Basin. (**A**) Map showing the location of the Sichuan Basin in China. (**B**) Map showing distribution of fold belts and the CK-1 well location in the southeastern Sichuan Basin. (**C**) Tectonostratigraphic framework showing intense tectonic deformation.

Shale gas content is one of the key parameters in the evaluation of shale gas reserves and development programs, and it determines the success of shale gas exploration^1^. China contains widely distributed shale gas resources^2^. Shale gas exploration has achieved remarkable success in the Sichuan Basin, China^3^. The Fuling shale gas field in the Sichuan Basin is the first commercial shale gas field in China, with a production capacity of more than 7.0 × 10^9^ m^3^ in 2017^4^. However, the Longmaxi Formation Shale has a high thermal evolution and complex multi-phase tectonic history, which can result in serious damage to shale gas reservoirs^5^. In many areas of the Sichuan Basin, the shale gas in the Longmaxi Formation has been totally lost^6,7^. For example, the lower Longmaxi Formation (TOC > 2%) has a similar reservoir quality in the Jiaoshiba, Qianjiang, and Youyang areas, but the gas content in the JY1 well in the Jiaoshiba area is approximately 3.14 m^3^/t, while the gas content in the QY1 well in the Qianjiang area is approximately 1.20 m^3^/t, and the gas content in the YC6 well in the Youyang area, which is located in the basin margin, is 0.01 m^3^/t (see Fig. 1 for location)^7^. Therefore, the desorption and loss of shale gas is common in the Sichuan Basin, and the desorption at the basin margin is particularly serious, as it controls whether the shale gas reservoir can be preserved today. Thus, it is meaningful to study the mechanism of shale gas desorption and to clarify the process of shale gas desorption and its controlling factors, which is significant to prospect the area where shale gas reservoirs are preserved.

Shale was previously identified as the source rock or cap rock for oil and gas reservoirs^8,9^. Thus, the shale sealing capacity (e.g., diffusion coefficient and breakthrough pressure) was the focus of many studies, as shale has not been regarded as a reservoir^10,11^. The shale gas content depends on the quantity of gas that is generated and displaced. The quantity of gas generated is controlled by the organic matter (OM) type, OM content, and OM maturity^12^. The quantity of gas displaced is mainly controlled by the diffusion coefficient and breakthrough pressure^11^.

With the large-scale development and utilization of shale gas, current knowledge about shale gas has changed^13^. It is recognized that shale gas is composed of adsorbed gas, free gas, and a small amount of dissolved gas^14^. The strong tectonic destruction of the Sichuan Basin reveals that shale gas desorption is one of the key factors controlling the successful exploration and development of shale gas in the Sichuan Basin^15,16^. However, knowledge of the shale gas desorption is lacking, as it is mostly based on physical simulation experiments^17,18^.

In the physical simulation experiments of the process of shale gas desorption, it was found that the velocity of shale gas desorption decreases gradually over time, the effect of temperature on shale gas desorption is obvious, and the desorption velocity at high temperatures is obviously higher than that at low temperatures^18^. In the early stage, the desorption content of shale gas is larger and the desorption velocity decreases over time^19^. The fractionation of gas components and isotopes during the shale gas desorption process are of greater concern^10,20^. The carbon isotope of alkane gets obviously heavier with desorption over time^21^. The CH_4_ content did not change much at different desorption stages, but C_2_H_6_, CO_2_, and N_2_ changed markedly^22^. The main reason for the change in gas composition is that the adsorption capacities of CH_4_, C_2_H_6_, CO_2_ and N_2_ in shale are different^23^. The adsorption capacity of shale is mainly controlled by the OM content^24^. The higher the OM content is, the stronger the shale adsorption capacity is.

However, knowledge about the capabilities and origin of shale gas desorption is lacking. Several crucial questions must be answered, such as how shale gas desorption happens, how intense shale gas desorption is, and whether the compositions of shale organic matter and minerals affect the shale gas desorption capability. On the one hand, slow shale gas desorption at the micro level over millions of years eventually leads to the disappearance of shale gas reservoirs. On the other hand, knowledge of the process of shale gas desorption and capability can be used to guide fracturing and exploit the resource more effectively.

Therefore, we designed a physical simulation experiment for the shale gas desorption process. Based on the analysis of the rapid desorption process of shale gas, we clarified the differences in shale gas desorption capability and the origin of shale gas desorption capability. The study is not only beneficial for revealing the microscopic mechanism of shale gas desorption but also for guiding shale gas exploration.

### Geological Background

The southeastern Sichuan Basin is an important part of the Upper Yangtze Plate. The Longmaxi Formation Shale mainly went through three major tectonic movements, including the Indosinian, Yanshan, and Himalayan Movements^25^. A series of NNE-trending faults and folds were formed in the southeastern Sichuan Basin due to the strong orogeny during the Yanshan Movement; then, a series of graben-horst faults were formed due to the release of crustal stress during the Himalayan Movement^26^. The southeastern Sichuan Basin is composed of the Jinfoshan, Qiyaoshan, Qianjiang, and Xiushan fold belts (Fig. 1). The strata ages in the southeastern Sichuan Basin are mainly Cambrian, Ordovician, Silurian, and Permian. The other strata were lost due to strong tectonic erosion^27^. There are two main sets of marine organic-rich shale distributed in the southeastern Sichuan Basin, namely, the lower Cambrian Niutitang Formation and the lower Silurian Longmaxi Formation^28^. The black shale of the Longmaxi Formation is regarded as the most important play for shale gas exploration and development in China^29^.

The Longmaxi Formation Shale is widely distributed in the southeastern Sichuan Basin; it is characterized by its shallow depth, large thickness, high thermal evolution, and high OM content^30^. The burial depth of the Longmaxi Formation Shale in most areas is less than 3400 m, and the formation is even uplifted to the surface in some areas^7^. The lithology of the Longmaxi Formation is dominated by carbonaceous shale, siliceous shale, clay shale, and silty shale^31^. The shale is rich in graptolites, and a few radiolarians and spicules also appear^32^. The lower section of the Longmaxi Formation formed in a deep shelf with a slow deposition rate, anoxic environment, and high OM content^33^. The upper section of the Longmaxi Formation gradually became a shallow shelf, and the OM content is generally less than 2.0%^34^.

## Material and Methods

### Samples

The samples were selected from the CK-1 well. The CK-1 well is located in the southeastern Sichuan Basin, where the depth of Longmaxi Formation ranges from 1087 m to 1166 m (Fig. 1). Because the gas in the shale can easily escape, the experiment was carried out in the field. The core sample from the drilling site was taken to simulate the shale gas desorption process directly, once the core was obtained from the subsurface. Pressure coring was used in the CK-1 well, which can effectively prevent the escape of shale gas during the coring process. In this experiment, 25 samples were selected from the bottom to the top of the Longmaxi Formation.

### Shale gas desorption test

A water bath was used to control the temperature during the shale gas desorption test. The gas volume was measured by acidified water displacement. The main materials required are the included desorption canister, water basin, gas valves, thermometer, pressure gauge, and measuring cylinder. The test temperatures in this study were 50 °C and 98 °C, with the former representing the reservoir temperature and the latter representing the highest temperature that the experimental setup can reach. The pressure coring was used during the coring process, which could prevent the gas from escaping^18^. The sample was heavier than 1.5 kg, and the time between getting the core to filling the canister was less than 3 min, which could reduce the measurement error. The shale gas content was transformed into STP (0 °C and 101 kPa).

**Figure 2:**
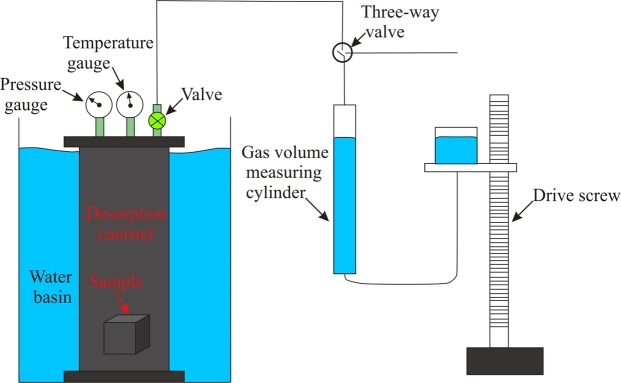
Experimental setup of the physical simulation of shale gas desorption process. The tests were carried out at 50 °C and 98 °C in succession.

The testing process is as follows: (a) prepare the instrument, e.g., record the local temperature and pressure at the well site, and add excess salt to the water basin to ensure that the salt water is saturated; (b) determine the leakage rate of the apparatus; (c) put the samples into the desorption canister with saturated salt water and record the time and sample information, such as the drilling time, core lifting time, reaching wellhead time, and sample depth; (d) put the desorption canister into the water bath with the thermostat set to 50 °C and record the time and gas volume until no gas comes out; (e) increase the temperature to 98 °C and record the time and gas volume until no gas comes out (Fig. 2).

### Geochemical tests

The total organic carbon (TOC) measurement was carried out on a CM 250 TOC analyzer. Each powder sample, which was approximately 200 mesh in size, was weighed to approximately 100 mg, and it was treated with 5% hydrochloric acid in the sample cell for 12 hours. After that, the sample was heated at 80 °C for 1 hour to remove carbonates. The mineral composition was determined using a Bruker D8 Advance X-ray diffractometer. The test conditions were Cu (monochrome), 40 kV, 30 mA, and scanning at a rate of 4°/min in the range of 3°~85°(2θ). The pore structure was observed by focused ion beam scanning electron microscope (FIB-SEM) and focused ion beam helium ion microscopy (FIB-HIM). The sample was polished for 1.5 h and 0.5 h at 6 kv and 4 kv, respectively, using a Hitachi IM4000 Ar ionizer. The maximum pixel resolution of the FIB-SEM is 0.8 nm, which was mainly used to observe the pores developed in minerals. The maximum pixel resolution of the FIB-HIM is 0.5 nm, which was mainly used to observe the pores developed in organic matter. The specific surface area was tested using a NOVA4000e automatic specific surface area tester. The accuracy is 0.01 m^2^/g and the minimum resolution relative pressure (P/P_0_) is 2 × 10^−5^ (N_2_). The specific surface area was calculated using the multi-point BET (Brunauer- Emmett- Teller) method. The maximum methane adsorption capacity was obtained using an isothermal adsorption tester. The test samples were 200-mesh powders. The shale gas composition was obtained using a Varian CP-3800 gas chromatograph.

## Results

### Shale gas content and composition characteristics

**Figure 3:**
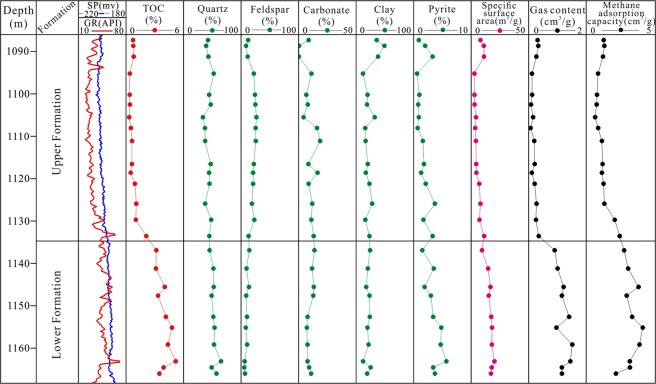
Well logs, TOC, mineral composition, specific surface area, gas content, and methane adsorption capacity of the CK1 well. The lower Formation has high gas content, with an average value of 1.19 m^3^/t. The upper Formation has low gas content, with an average value of 0.20 m^3^/t. The TOC, specific surface area, and maximum methane adsorption capacity of the lower Formation are markedly higher than those of the upper Formation. The mineral compositions (especially quartz and clay) of the upper Formation and the lower Formation are similar.

The shale gas content of the Longmaxi Formation in the CK-1 well is high, ranging from 0.07 m^3^/t to 1.52 m^3^/t, with an average value of 0.60 m^3^/t (Fig. 3 and Table 1). The Longmaxi Formation Shale can be divided into two parts: the upper Formation and the lower Formation, based on the vertical gas content in the Longmaxi Formation. As shown in Fig. 3, the gas content of the upper Formation (from sample CK1-1 to sample CK1-15) is obviously less than that of the lower Formation (from sample CK1-16 to sample CK1-25). The reason of the difference of gas content between the upper Formation and lower Formation is that the lower Longmaxi formation was formed in the deep-water shelf facies with rich organic matter shale, while the upper Longmaxi Formation was formed in the shallow-water shelf facies with organic-poor shale^35,36^. The shale gas content of the upper Formation is 0.07 m^3^/t–0.35 m^3^/t, with an average value of 0.20 m^3^/t, while the shale gas content of the lower Formation is 0.90 m^3^/t–1.52 m^3^/t, with an average value of 1.19 m^3^/t. Therefore, in this study, the characteristics and origin of shale gas desorption are discussed based on the comparison of the upper Formation and the lower Formation.

**Table 1:** Shale gas content, gas composition, TOC content and minerals composition of the Longmaxi Formation in the southeastern Sichuan Basin.

Formation	Sample ID	Depth	Gas content	Gas composition (%)					TOC	Specific surface area	Quartz	Feldspar	Carbonate	Pyrite	Clay
		(m)	(m^3^/t)	CH_4_	N_2_	CO_2_	C_2_H_6_	C_3_H_8_	(%)	(m^2^/g)	(%)	(%)	(%)	(%)	(%)
Upper Formation	CK1-1	1087.32	0.3099	93.75	4.50	0.93	0.81	0.01	0.74	8.05	42.5	12.2	8.7	0.9	35.7
	CK1-2	1088.63	0.3351	92.77	5.81	0.64	0.77	0.01	0.76	10.97	39.3	8.7	0.4	2.0	49.3
	CK1-3	1091.18	0.2670	92.09	6.96	0.35	0.59	0.01	0.80	11.19	43.2	11.5	0.2	3.3	38.1
	CK1-4	1095.20	0.1149	85.47	11.66	0.97	1.85	0.05	0.40	2.84	52.5	22.2	11.2	0.6	12.0
	CK1-5	1100.22	0.1212	93.42	5.30	0.62	0.65	0.01	0.39	2.86	44.5	24.2	6.7	1.0	19.9
	CK1-6	1102.50	0.0816	85.93	11.89	1.68	0.49	0.01	0.41	2.99	47.3	24.9	8.0	0.8	19.0
	CK1-7	1105.48	0.1522	89.19	8.91	1.13	0.75	0.01	0.33	4.19	33.7	26.7	4.3	0.9	32.4
	CK1-8	1108.03	0.0658	62.37	36.03	1.23	0.35	0.01	0.51	3.53	37.2	25.3	16.0	0.7	15.9
	CK1-9	1111.10	0.1952	89.53	7.84	1.46	1.14	0.03	0.63	4.14	37.6	25.6	18.7	1.6	16.5
	CK1-10	1116.55	0.2092	91.05	5.40	2.04	1.48	0.01	0.62	4.38	47.2	22.2	8.5	1.8	20.3
	CK1-11	1118.61	0.1106	84.99	13.67	0.73	0.61	0.01	0.49	4.57	44.4	20.9	16.5	1.3	16.9
	CK1-12	1121.20	0.2088	89.48	9.06	0.83	0.62	0.01	0.93	7.03	45.2	20.9	8.7	2.1	23.1
	CK1-13	1125.90	0.2737	94.00	4.36	0.96	0.67	0.01	1.07	8.06	37.6	18.9	11.9	3.7	27.9
	CK1-14	1129.70	0.2536	91.98	6.70	0.75	0.57	0.01	1.02	7.21	48.1	23.1	11.1	1.7	16.0
	CK1-15	1133.55	0.3486	87.44	11.31	0.60	0.64	0.01	2.11	11.29	44.8	13.1	13.1	3.3	23.2
Lower Formation	CK1-16	1136.91	0.9032	95.32	3.6	0.47	0.61	0.01	3.16	9.42	45.2	14.3	13.8	1.5	24.3
	CK1-17	1141.20	1.0043	95.18	3.46	0.71	0.64	0.01	3.12	14.76	52.0	9.5	11.7	3.5	20.4
	CK1-18	1145.55	1.2144	95.02	3.95	0.43	0.59	0.01	4.05	16.77	52.5	13.7	13.2	1.9	17.5
	CK1-19	1147.62	1.1540	94.73	4.05	0.58	0.63	0.01	3.34	15.26	48.8	8.9	12.8	3.0	23.3
	CK1-20	1152.60	1.4061	96.08	2.85	0.41	0.65	0.01	4.16	17.45	51.8	10.3	7.8	3.4	23.8
	CK1-21	1155.13	0.9648	94.31	4.40	0.59	0.68	0.01	4.80	18.20	53.9	8.2	7.2	4.8	19.7
	CK1-22	1159.17	1.517	96.28	2.66	0.41	0.64	0.01	4.37	17.99	53.0	10.5	7.8	4.7	21.7
	CK1-23	1163.11	1.4486	95.66	3.36	0.34	0.63	0.01	5.18	20.24	65.2	6.3	7.8	5.7	12.1
	CK1-24	1164.55	1.1542	94.87	2.80	1.12	1.20	0.02	3.92	17.86	48.9	5.6	9.2	3.4	25.2
	CK1-25	1166.00	1.1558	95.79	2.52	0.75	0.92	0.01	3.48	17.15	57.3	6.9	10.9	3.7	19.2

The composition of the shale gas is mainly CH_4_, with minor N_2_, CO_2_, etc. The shale gas content was in STP (0 °C and 101kPa).

The average content of CH_4_ is 91.1%, N_2_ is 7.32%, CO_2_ is 0.83%, C_2_H_6_ is 0.76%, and C_3_H_8_ is 0.01% (Table 1). The average CH_4_ content in the upper Formation is significantly lower than that in the lower Formation, i.e., 88.23% and 95.32%, respectively. The average N_2_ content in the upper Formation is higher than that in the lower Formation, i.e., 9.96% and 3.37%, respectively. The contents of CO_2_, C_2_H_6_ and C_3_H_8_ in the upper Formation and the lower Formation are all lower than 1.0%.

### Shale geochemical characteristics

The TOC of the lower Formation is 3.12% ~ 5.18%, with an average value of 3.96%, while the TOC of the upper Formation ranges from 0.33% to 2.11%, with an average value of 0.75% (Fig. 3). For the specific surface area, the mean value of the lower Formation is 16.51 m^2^/g, while the mean value of the upper Formation is 6.22 m^2^/g. The maximum methane adsorption capacity of the lower Formation is higher than that of the upper Formation, with mean values of 3.36 m^3^/t and 0.98 m^3^/t, respectively (Fig. 3).

**Figure 4:**
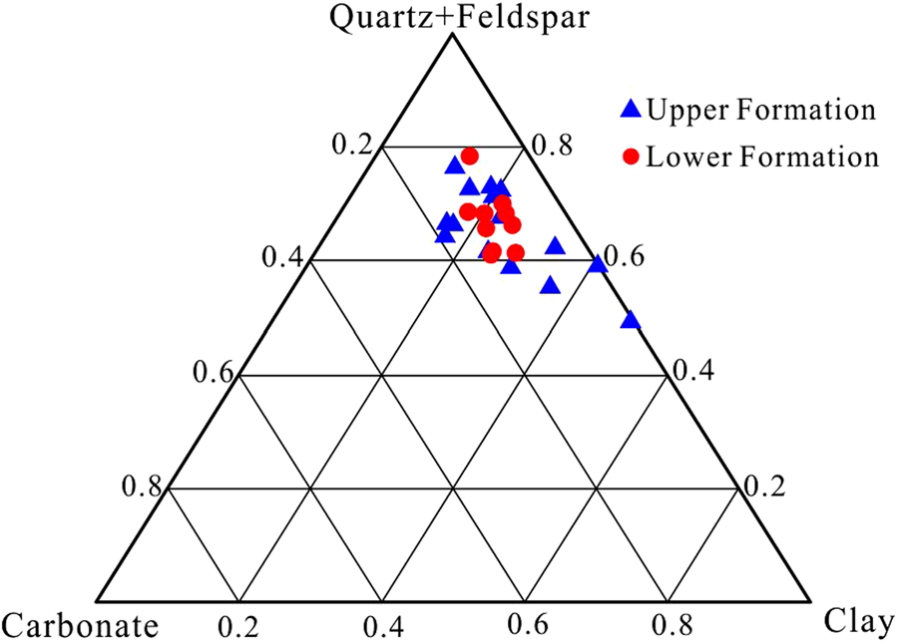
Main mineral composition of the Longmaxi Formation Shale in the CK-1 well. The minerals in the lower Formation are more concentrated, and the composition changes slightly. The quartz + feldspar and clay minerals vary widely, and the carbonate content changes relatively slightly in the upper Formation.

The mineral composition of the Longmaxi Formation Shale is mainly quartz and feldspar, with an average value of 47.0%. Following these is clay minerals, with an average content of 22.9%. The carbonate minerals are the lowest, with a content of less than 20% (Fig. 4). The quartz content in the upper Formation shale is lower than that in the lower Formation. The feldspar content in the upper Formation is clearly higher than that in the lower Formation, with mean values of 20.0% and 9.4%, respectively. The clay contents of the upper Formation and the lower Formation are similar, i.e., 24.4% and 20.7%, respectively. The pyrite content of the upper Formation is 1.7%, whereas that of the lower Formation is 3.6%.

### Shale gas desorption process characteristics

**Figure 5:**
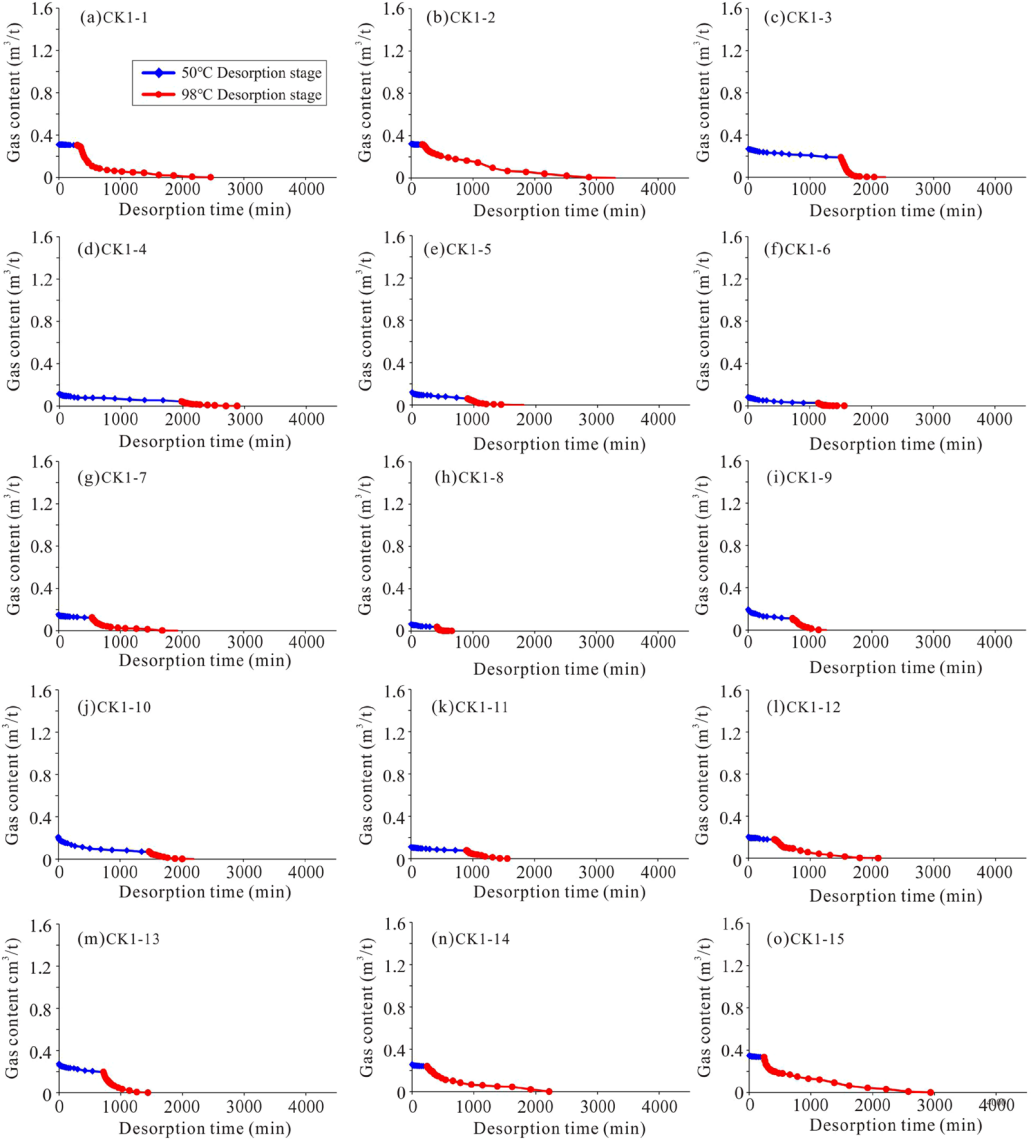
The shale gas desorption process of the upper Formation (sample CK1-1 to sample CK1-15) in the CK-1 well. Same coordinate axes are used for comparison in Figs 5 and 6.

**Figure 6:**
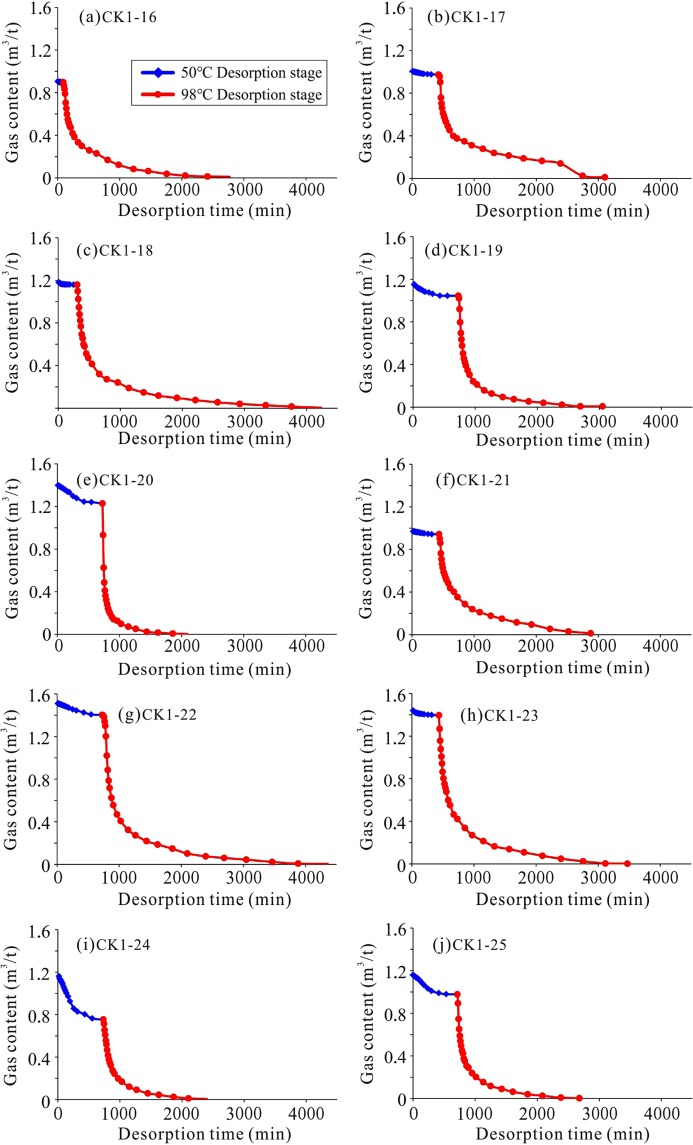
The shale gas desorption process of the lower Formation (sample CK1-16 to sample CK1-25) in the CK-1 well. The gas content at the 98 °C desorption stage is obvious higher than at the 50 °C desorption stage.

The shale gas desorption process is different for each sample (Figs 5 and 6). It can be seen that the gas content of the lower Formation is several times higher than that of the upper Formation, but their desorption times are similar. For example, the gas content of sample CK1-1 is 0.31 m^3^/t, and it lost 83% of the total gas within 1000 min. The gas content of sample CK1-23 is 1.45 m^3^/t, and it lost 82% of the total gas in the first 1000 min. The main reason for this is that shale (sample CK1-16 to sample CK1-25) with high gas content can lose most of its gas quickly and early in the process. In addition, the effect of temperature on desorption is obvious. Most of the samples have a much lower gas loss at 50 °C than at 98 °C. The upper Formation lost 2 ~ 67% of the total gas content at 50 °C, with an average value of 31%. In contrast, the lower Formation lost 1 ~ 35% of the total gas content at 50 °C, with an average value of 9%.

**Figure 7:**
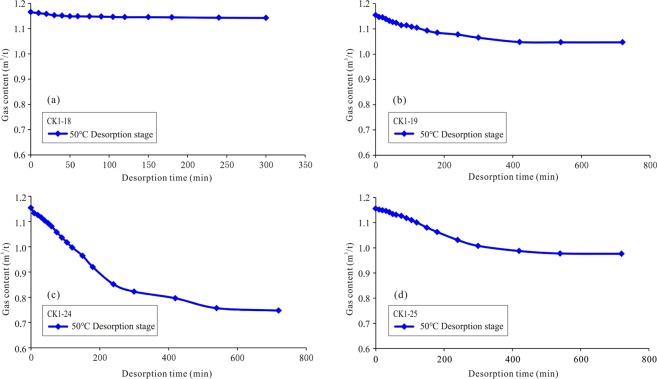
Shale gas desorption curves of samples CK1-18, CK1-19, CK1-24, and CK1-25 at the 50 °C desorption stage (the integrated desorption process of these samples is shown in Fig. 6). The initial gas content of the four samples is similar. The CK1-18 sample lost the least gas, i.e., only 0.02 m^3^/t. The CK1-19 sample lost 0.11 m^3^/t. The CK1-24 sample experienced heavy gas loss, at 0.41 m^3^/t. The CK1-25 sample lost 0.18 m^3^/t.

**Figure 8:**
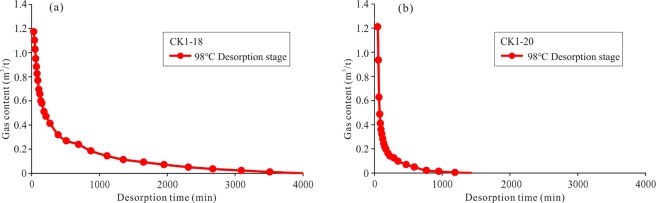
Shale gas desorption curves of samples CK1-18 and CK1-20 at the 98 °C desorption stage (the integrated desorption process of the samples is shown in Fig. 6). The initial gas contents of the two samples are similar. The CK1-18 sample took 4000 min to experience loss. The CK1-20 sample took 1500 min to experience loss, while most of the gas was lost in the initial 200 min.

Based on the shapes of the desorption curves, it can be seen that although the initial gas content of the samples is similar at the 50 °C desorption stage, the desorption curves are different (Fig. 7). For example, the initial gas contents of samples CK1-18, CK1-19, CK1-24, and CK1-25 are similar, i.e., nearly 1.2 m^3^/t, but the desorption curves of the four samples are obviously different. Sample CK1-18 experienced a slight loss, while sample CK1-24 experienced a heavy loss (Fig. 7). Similarly, the shale gas desorption curves are different at 98 °C. For example, the initial gas contents of sample CK1-18 and sample CK1-20 are close to 1.2 m^3^/t at 98 °C, but sample CK1-18 took nearly 4000 min to lose its gas content, while sample CK1-20 only took approximately 1500 min. The desorption of sample CK1-18 is a relatively slow process, while that of sample CK1-20, which lost 90% of its gas in the first 200 min, is a rapid loss process (Fig. 8). Therefore, for samples with similar temperatures and initial gas contents, variations in their shale properties are responsible for causing these different desorption processes.

## Discussion

### Shale gas desorption capability

The shale samples in this study have different desorption processes; the reason for this difference is that the shale samples have different desorption abilities. Therefore, it is necessary to define the desorption capability of shale gas. The shale gas desorption capability is determined by temperature, pressure, and shale properties. In this experiment, the main shale gas desorption experiment was done at 98 °C. Thus, in this paper, we mainly analyze the shale gas desorption capability at 98 °C. The pressure in this experiment changes with the shale gas content, which is consistent with the state equation of an ideal gas (Equation 1). According to Equation 1, the pore volume in the samples is unchanged, the temperature is 98 °C, and R is the ideal gas constant. Thus, pressure and the gas content have a positive linear correlation. The shale desorption velocity is controlled by the shale gas content under the limitations of the experimental conditions^18^. Therefore, in this study, the desorption capability is defined as a function of the desorption velocity and the gas content (Equation 2).

1 $${\rm{PV}}={\rm{NRT}}$$

where P is pressure in Pa, V is volume in m^3^, N is quantity in mol, R is the ideal gas constant, and T is temperature in K.

2 $${{\rm{A}}}_{{\rm{desorption}}}={\rm{f}}({{\rm{V}}}_{{\rm{desorption}}}/{\rm{N}})$$

where A_desorption_ is the desorption capability, V_desorption_ is the desorption velocity, and N is the gas content.

**Figure 9:**
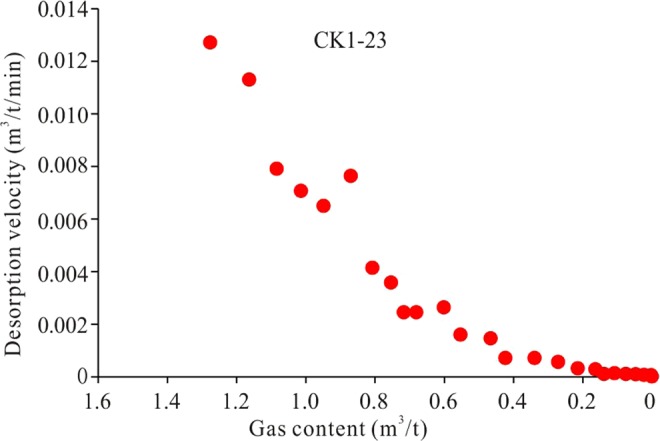
Relationship between shale gas desorption velocity and gas content at the 98 °C desorption stage, using sample CK1-23 as an example. With decreasing gas content, the desorption velocity is reduced.

Shale gas desorption velocity can be obtained based on the variations in gas content over time (Fig. 9). For example, the desorption velocity of sample CK1-23 is approximately 0.013 m^3^/t/min at the beginning. With decreasing gas content, the desorption velocity gradually decreases until all of the gas is lost.

**Figure 10:**
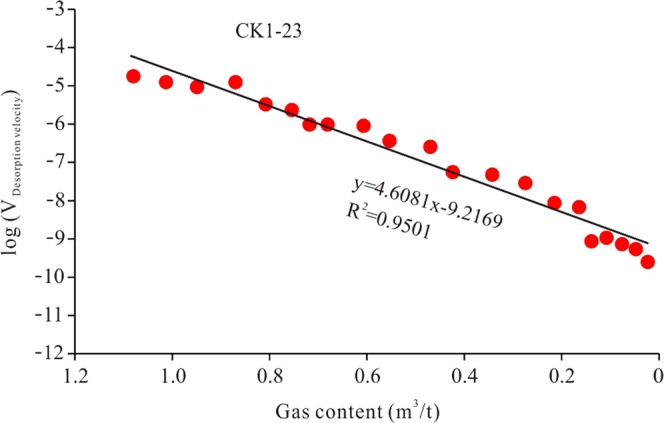
Calculation of shale gas desorption capability, using sample CK1-23 as an example. The slope of the line between log (V_desorption_) and gas content is a constant value, which represents the intrinsic properties of the shale. The slope can be used to represent the shale gas desorption capability, which is a quantitative value.

The shale gas desorption velocity was obtained using a logarithm and then making a scatter plot with the shale gas content (Fig. 10). Figure 10 shows that there is a good linear relationship between log (V_desorption_) and gas content, with correlation coefficients of up to 0.9501. Thus, the slope of the linear relationship represents the shale properties, which is the comprehensive parameter reflecting desorption velocity and gas content. Therefore, the slope can represent the shale desorption capability. As shown in Fig. 10, the desorption capability of sample CK1-23 is 4.6081.

The shale gas desorption abilities of the 25 samples were obtained based on this method (Table 2), and their correlation coefficients (R^2^) are mostly higher than 0.7. Therefore, the desorption capability is a relatively reliable value. The shale gas desorption capability clearly varies within the Longmaxi Formation. The desorption capability of the lower Formation is low, generally less than 10. However, the upper Formation has a high desorption capability, which is generally higher than 10. This is one important reason why the shale gas content of the lower Formation is higher than that of the upper Formation. The Longmaxi Formation Shale is high-over mature shale, and no more gas is being generated because the formation is in a stage of uplift, leading to the desorption process in the shale gas reservoir^37^. The shale gas content of the lower Formation is higher than that of the upper Formation, indicating that the lower Formation shale has stronger gas storage capability (in other words, a weaker gas desorption capability), which can effectively prevent the gas from migrating to the overlying strata^38^. As the upper Formation has a weaker desorption capability than the lower Formation, the gas content of the upper Formation may never be higher than that of the lower Formation.

**Table 2:** Shale gas desorption capability and desorption proportions at 50 °C and 98 °C desorption stages.

Formation	Sample ID	50 °C desorption stage		98 °C desorption stage		Desorption capability at 98 °C	Correlation coefficient (R^2^)
		Desorption content (m^3^/t)	Desorption proportion (%)	Desorption content (m^3^/t)	Desorption proportion (%)		
Upper Formation	CK1-1	0.0061	1.97	0.3039	98.03	19.915	0.9046
	CK1-2	0.0055	1.72	0.3155	98.28	8.9957	0.7641
	CK1-3	0.0780	29.22	0.1890	70.78	9.9803	0.6666
	CK1-4	0.0716	62.28	0.0434	37.72	68.253	0.6863
	CK1-5	0.0606	50.09	0.0604	49.91	45.549	0.7544
	CK1-6	0.0548	66.84	0.0272	33.16	275.74	0.6510
	CK1-7	0.0279	18.35	0.1241	81.65	41.838	0.8160
	CK1-8	0.0286	43.29	0.0374	56.71	101.58	0.7688
	CK1-9	0.0848	43.51	0.1102	56.49	19.832	0.7125
	CK1-10	0.1386	66.31	0.0704	33.69	32.817	0.7646
	CK1-11	0.0335	30.19	0.0775	69.81	35.901	0.6261
	CK1-12	0.0258	12.63	0.1782	87.37	15.433	0.7207
	CK1-13	0.0782	28.54	0.1958	71.46	17.363	0.8969
	CK1-14	0.0180	6.97	0.2410	93.03	13.612	0.6465
	CK1-15	0.0133	3.81	0.3357	96.19	12.84	0.7862
Lower Formation	CK1-16	0.0091	1.01	0.8939	98.99	6.7794	0.9598
	CK1-17	0.0324	3.23	0.9706	96.77	8.0119	0.9763
	CK1-18	0.0228	1.92	1.1632	98.08	6.462	0.9431
	CK1-19	0.1074	9.30	1.0466	90.70	7.7613	0.9444
	CK1-20	0.1713	12.19	1.2347	87.81	9.1802	0.9488
	CK1-21	0.0269	2.79	0.9381	97.21	6.3003	0.9756
	CK1-22	0.1080	7.12	1.4090	92.88	5.8831	0.9574
	CK1-23	0.0452	3.12	1.4038	96.88	4.6081	0.9501
	CK1-24	0.4067	35.24	0.7473	64.76	7.954	0.9842
	CK1-25	0.1797	15.54	0.9763	84.46	6.9513	0.9545

The lower Formation lost gas mainly at the 98 °C desorption stage. The upper Formation lost gas at both stages, with relatively small differences. The desorption capability of the lower Formation is obviously lower than that of the upper Formation.

**Figure 11:**
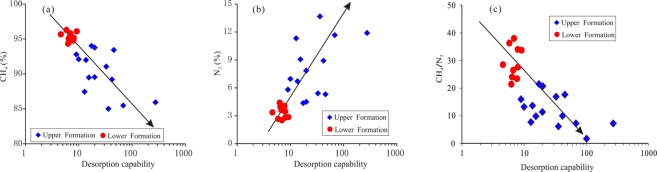
The relationship between desorption capability and N_2_, CH_4_ and CH_4_/N_2_. As the desorption capability increases, the N_2_ percentage increases, while the CH_4_ percentage and CH_4_/N_2_ decreases.

The shale gas composition can reflect the sealing capability of the shale. The lower the N_2_ percent is, the lower the amount of exchange between the shale gas and the outside is. Although the origin of N_2_ in the shale is still controversial, there are some hypotheses, such as atmospheric exchange, mantle supply, and hydrocarbon source rock generation^39–41^. If the N_2_ was generated by hydrocarbon source rock, the proportion of N_2_ in the lower Formation should be consistent with that in the upper Formation, which is not the case in this study (Table 1). Therefore, the source of N_2_ in the Longmaxi Formation may not come from hydrocarbon source rock generation. The nitrogen isotopes in the Lower Cambrian Niutitang Formation which below the Longmaxi Formation in the study area is ranging from −2.6‰ to 0‰, indicating N_2_ is from the atmosphere^42,43^. Therefore, the source of N_2_ in the Longmaxi Formation is more likely to come from the atmosphere or the deep earth. The N_2_ could be transported into the shale, indicating that the shale has the capability to receive external gas. The higher the N_2_ percentage is in the shale gas from the same area and formation, the stronger the receiving capability of the shale is, indicating a stronger desorption capability for the shale. It can be seen from Fig. 11 that the N_2_ percentage in the lower Formation is low with a weak desorption capability. In addition, with increasing desorption capability, the N_2_ percentage increases. Correspondingly, the lower the desorption capability is, the higher the proportion of CH_4_ is. Therefore, the desorption capability can be used to indicate the desorption characteristics of the shale.

### Origin of shale gas desorption

**Figure 12:**
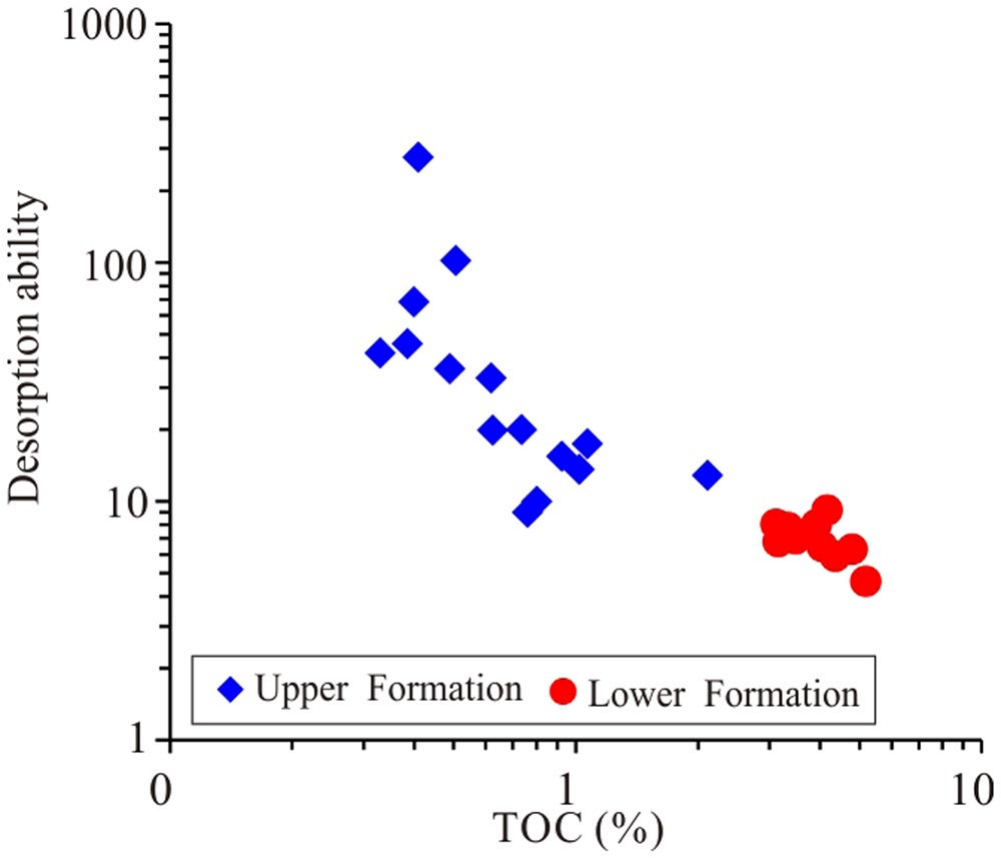
Relationship between shale gas desorption capability and TOC. The desorption capability decreases with increasing TOC. The desorption capability of the lower Formation, which has high TOC, is weaker than that of the upper Formation, which has low TOC.

**Figure 13:**
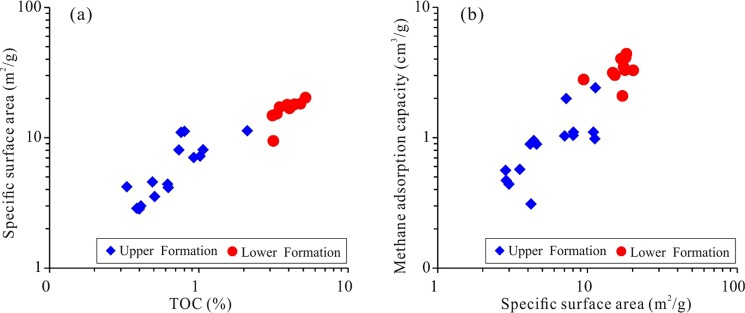
Relationship between specific surface area, TOC, and maximum methane adsorption capacity. The specific surface area increases with increasing TOC, and the maximum methane adsorption capacity increases with increasing specific surface area.

**Figure 14:**
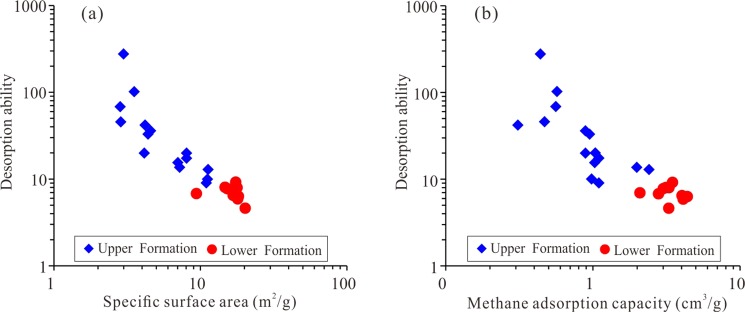
Relationship between shale gas desorption capability, specific surface area, and maximum methane adsorption capacity. The desorption capability has a negative correlation with both the specific surface area and the maximum methane adsorption capacity. The lower Formation, which has high TOC, has a higher specific surface area and maximum methane adsorption capacity than the upper Formation, leading to the lower Formation having a low desorption capability.

**Figure 15:**
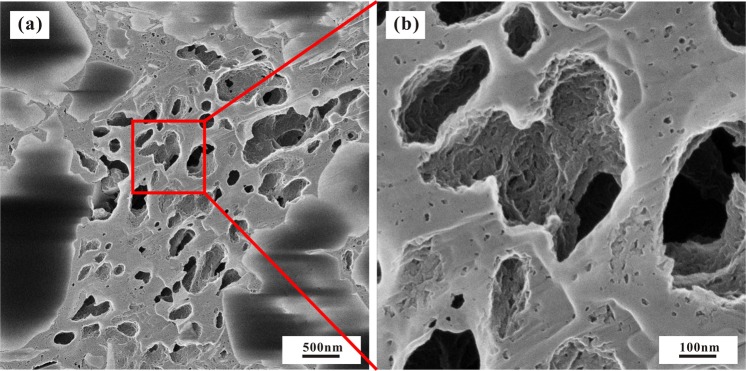
Characteristics of the OM pores of the Longmaxi Formation Shale. (**a**) The OM pores are well-developed. (**b**) Many small OM pores developed in the large OM pores, implying a high specific surface area.

Shale is mainly composed of various minerals and OM^44^. Although the OM content in the shale is relatively low, ranging from 0.5% to 5.1%, the effect of OM on shale gas desorption is intense. The desorption capability of high-TOC shale can be tens of times higher than that of low-TOC shale. The shale gas desorption capability decreases with increasing OM content, indicating that OM can control shale desorption capability (Fig. 12). For example, the desorption capability of sample CK1-6, which contains 0.41% TOC, is as high as 275.74, while the desorption capability of sample CK1-23, which contains 5.18% TOC, is only 4.61, and the difference between them is approximately 60 times. The OM in shale generally has a high specific surface area, and a higher specific surface area also leads to a stronger adsorption capacity^24^. For the Longmaxi Formation Shale, the OM content has a strong positive correlation with the specific surface area, as does the specific surface area with the maximum methane adsorption capacity (Fig. 13)^24^. It can be implied that the shale has a high specific surface area and methane adsorption capacity and that CH_4_ cannot easily migrate out of the shale, showing a weak desorption capability (Fig. 14). The FIB-HIM photos clearly show that the OM in the shale has developed many pores and that the OM pores have a complex structure, as their macropores and mesopores are filled with micropores and these pores are well-connected with each other (Fig. 15)^45^. Therefore, these nanoscale pores have higher specific surface area and developed an intricate pore system, which reduces the shale gas desorption capability.

**Figure 16:**
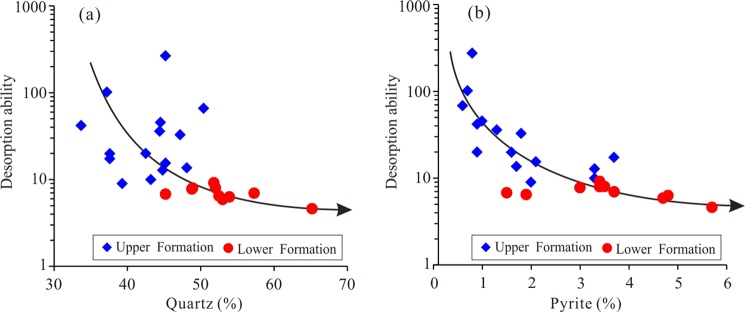
Relationship between shale gas desorption capability, quartz, and pyrite. The desorption capability decreases with increasing quartz content and pyrite content.

**Figure 17:**
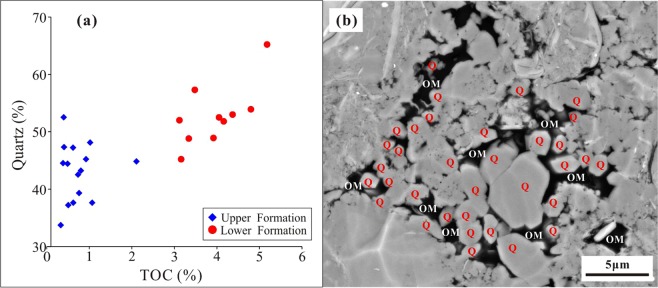
Relationship between quartz content and TOC. (**a**) The quartz content increases with increasing TOC. (**b**) OM and quartz are generally developed together.

Compared with the OM, the minerals in the shale have a relatively weak influence on shale gas desorption capability, although minerals are the main component of the shale. The desorption capability has a weak negative correlation with quartz content (Fig. 16), which may be because the quartz is mainly biogenic and coupled to the OM^46^. The OM and quartz have a positive correlation (Fig. 17). The quartz content increases with increasing OM content due to the biogenic quartz carries a lot of organic matter^47,48^. The shale gas desorption capability is negatively correlated with OM, which leads to its negative correlation with quartz content. In addition, the FIB-SEM photos show that the quartz has developed few pores (Fig. 17b), and it is difficult for the few pores to provide a large storage space for shale gas^49^, resulting in only a small contribution to the shale gas desorption. Similarly, pyrite is coupled with OM^50^. Therefore, the shale gas desorption capability is negatively correlated with OM, which leads to a negative correlation between the shale gas desorption capability and pyrite (Fig. 16b).

**Figure 18:**
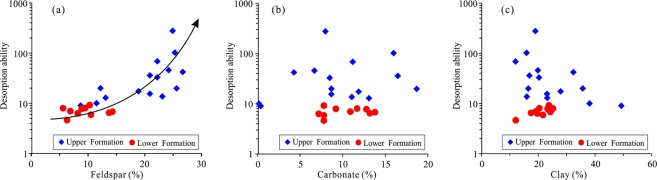
Relationship between shale gas desorption capability, feldspar, carbonate minerals, and clay minerals. The desorption capability increases with increasing feldspar. There is no correlation between desorption capability and carbonate or clay.

**Figure 19:**
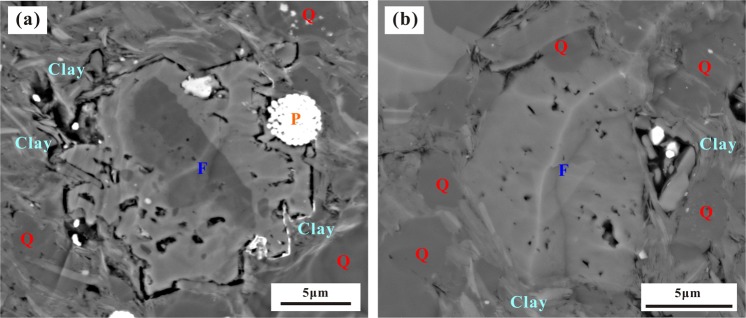
Characteristics of feldspar pores in the Longmaxi Formation Shale. (**a**) Pores in K-feldspar. (**b**) Pores in albite.

The shale gas desorption capability has a strong positive correlation with feldspar content (Fig. 18a). The major reason for this is probably because feldspar develops pores well in the shale (Fig. 19) but has little effect on methane adsorption^51^. The result is that the CH_4_ stored in the feldspar pores is more easily migrated and lost; as more feldspar pores develop, the more easily CH_4_ is migrated and lost. Therefore, not all pores are beneficial to the shale gas desorption capability. Pores in OM will lead to increased specific surface area and adsorption capacity^52^, which weaken the shale gas desorption capability. The pores in minerals can only provide gas storage space and have little effect on enhancing the adsorption capacity; thus, the more pores are connected, the stronger the desorption capability of shale gas is. In addition, there is no obvious correlation between desorption capability, carbonate minerals, and clay minerals (Fig. 18b,c), which indicates that carbonate and clay have no obvious controlling effect on the shale gas desorption capability.

## Conclusions

The shale gas desorption process is mainly controlled by temperature, pressure, and shale properties. The desorption content at the 50 °C desorption stage is clearly less than that at the 98 °C desorption stage. The gas content of the lower Formation is significantly higher than that of the upper Formation, but they take nearly the same time to lose all their respective gas contents. The shale with higher initial gas content has faster desorption at the early stage, and most of the gas is lost within a short time.

The shale gas desorption capability of the Longmaxi Formation exhibits obvious differences. The lower Formation has a low desorption capability, which generally falls below 10, causing it to currently contain high gas contents. However, the upper Formation has a high desorption capability, which is generally higher than 10, resulting in its currently relatively low gas content. The gas content of the upper Formation may never be higher than that of the lower Formation due to its high desorption capability.

The OM is the key factor in determining the shale gas desorption capability of the Longmaxi Formation Shale with high thermal evolution. The high OM content leads to a high specific surface area and a strong methane adsorption capacity of the shale. Thus, combined with the intricate OM pore structure, the shale exhibits a weak desorption capability. The feldspar content can increase the shale gas desorption capability, as feldspar pores are large and their connectivity is good, which is beneficial for the desorption and loss of shale gas. Other minerals, such as quartz, pyrite, carbonate, and clay, exert no obvious effect on the shale gas desorption capability.
